# Dielectrical Properties of CeO_2_ Nanoparticles at Different Temperatures

**DOI:** 10.1371/journal.pone.0122989

**Published:** 2015-04-24

**Authors:** Reza Zamiri, Hossein Abbastabar Ahangar, Ajay Kaushal, Azmi Zakaria, Golnoosh Zamiri, David Tobaldi, J. M. F. Ferreira

**Affiliations:** 1 Department of Physics, Faculty of Science, Universiti Putra Malaysia, 43400 UPM Serdang, Selangor, Malaysia; 2 Department Materials and Ceramic Engineering (DEMaC), CICECO, University of Aveiro, Campus Santiago, 3810-193, Aveiro, Portugal; 3 Department of Chemistry, Faculty of Science, Islamic Azad University, Najafabad Branch, Najafabad, Isfahan, Iran; Washington State University, UNITED STATES

## Abstract

A template-free precipitation method was used as a simple and low cost method for preparation of CeO_2_ nanoparticles. The structure and morphology of the prepared nanoparticle samples were studied in detail using X-ray diffraction, Raman spectroscopy and Scanning Electron Microscopy (SEM) measurements. The whole powder pattern modelling (WPPM) method was applied on XRD data to accurately measure the crystalline domain size and their size distribution. The average crystalline domain diameter was found to be 5.2 nm, with a very narrow size distribution. UV-visible absorbance spectrum was used to calculate the optical energy band gap of the prepared CeO_2_ nanoparticles. The FT-IR spectrum of prepared CeO_2_ nanoparticles showed absorption bands at 400 cm^-1^ to 450 cm^-1^ regime, which correspond to CeO_2_ stretching vibration. The dielectric constant (ε_r_) and dielectric loss (tan δ) values of sintered CeO_2_ compact consolidated from prepared nanoparticles were measured at different temperatures in the range from 298 K (room temperature) to 623 K, and at different frequencies from 1 kHz to 1 MHz.

## Introduction

Nanomaterials have attracted considerable attention in a broad range of advanced applications in multidisciplinary fields including material science and biology, based on the chemical composition, size, shape and surface dependent properties of nanoparticles [1,2]. It is well known that material properties changes when (i) particle size reduces to nano-scale or (ii) materials transferred to nanostructures form, leading to recent advances in synthesis of nanostructures [3]. The particle size of materials affects their basic properties such as lattice symmetry, cell parameters, and structural characteristics. Generally, phases in bulk form are unstable in bulk material due to high surface energy, however the surface energy decreases rapidly when size reduces to nanoscale level leading to high stability of materials at nanostructure levels [3,4].

Cerium (Ce) is one of the most abundant rare earth elements of the lanthanide series in earth’s crust and is present at about 66 ppm as a free metal and/or oxide forms. The two main oxidation sates of cerium are ceric- Ce (IV) and cerous- Ce (III), and can auto regenerate ($\varepsilon^{0}\frac{Ce\left(IV\right)}{Ce\left(III\right)}=\text{1.76}$) [5]. Both oxidation sates of Ce show strong absorbance peaks in ultraviolet light wavelength in the range at ~ 230–260 nm and at ~ 300–400 nm corresponding to Ce (III) and Ce (IV) oxidation states of cerium, respectively [6]. Materials containing Ce are widely used in the field of metallurgy, ceramics, and smart glasses and in optics. Cerium oxide nanoparticles (CNPs) have been widely utilized in various advanced technologies, such as solid-oxide fuel cells, high-temperature oxidation protection materials, catalytic materials, oxygen sensors and solar cells, etc [7–9]. When the size of cerium oxide reduces to nanometre scale, it shows exceptional properties including increase of optical band gap of nanoceria as a result of the quantum size effect at nanometre scale. Nanoparticle form of cerium shows remarkable catalytic reactivity due to high mobility of surface oxygen vacancies [10]. These oxygen vacancies alter the electronic and valence arrangement, which fix the oxidation state. CeO_2_ nanoparticles have also been used as a free radical scavenger, to modulate oxidative stress in biological system [11]. For example, CeO_2_ nanoparticles are able to rescue HT22 cells from oxidative stress-induced cell death, and protect normal human breast cells from radiation-induced apoptotic cell death [11,12]. In fluorite structure, CeO_2_ has a stable structure over a wide range of temperature in its pure stoichiometric form and the structure remains unchanged. Furthermore, CeO_2_ has also been regarded as a possible gate dielectric material in metal-oxide-semiconductor and memory devices for next generation devices [13–15]. This is because CeO_2_ has high ability for oxygen storage which makes CeO_2_ as one of the most important automobile exhaust catalysts [16].

Various chemical route methods have been reported on the synthesis of CeO_2_ oxide nanoparticles such as reversed micelles route, co-precipitation, hydrothermal synthesis, forced hydrolysis, solvo-thermal synthesis, sol–gel process, pyrolysis, electrochemical method, and sono-chemical methods [17–26]. In this work, we report on synthesis of CeO2 nanoparticles by a facile and single one-pot template-free precipitation route without any need of capping ligands and other additives. The structural, optical and dielectric properties of the prepared CeO_2_ nanoparticles samples have been systematically investigated.

## Material and Methods

### Synthesize and characterization of CeO_2_ nanoparticles

CeO_2_ nanoparticles were prepared by a wet chemical precipitation method. Firstly, (30 mmol) of Ce(NO_3_)_3_.6H_2_O (Aldrich, Germany) was dissolved in distilled water. The dissolved solution was then added drop wise into a beaker containing 100 mL of 0.4M NaOH (Merck, Germany) solution at room temperature. The pH value of the solutions during experiment was maintained constant to around 13. At this pH, precipitates immediately formed and were then ultra-centrifuged at a speed of 10,000 rpm for 10 min to obtain clear supernatant liquids separated from precipitates. The centrifuged precipitates were further washed several times with distilled water to complete removal of unwanted Na^+^, Cl^-^, NO_3_^-^ ions. The washed precipitates were then dried at 353 K for 3 h.

The crystallinity of the prepared nanoparticles samples was studied by measuring powder X-ray diffraction (XRD) pattern using Panalytical X’Pert Pro, NL diffractometer in θ/θ geometry, equipped with a fast RTMS detector, with Cu Kα (1.54 Å) radiation (45 kV and 40 mA, 2θ range from 20–125° with a virtual step scan of 0.1° and virtual time per step of 500 s). The incident beam pathway was: 0.5° divergence slit, 0.5° anti-scattering slit, 0.04 rad soller slits, and a 15 mm copper mask. The pathway of the diffracted beam included a Ni filter, soller slits (0.04 rad), and an anti-scatter blade (5 mm).

The microstructure topography and chemical analysis of the samples was studied using scanning electron microscopy (SEM, SU-70, Hitachi) and by measuring in-situ energy-dispersive X-ray spectroscopy (EDS) patterns, respectively. To prevent the charge build up during SEM observations, samples were coated with carbon. HITACHI SU-70 high resolution transmission electron microscope (HRTEM), equipped with a Bruker EDS detector was used to further analyses of the structure of the prepared nanoparticles samples. The optical properties of the samples were measured by using UV–Visible–NIR (Perkin-Elmer, Lambda 35), Raman (laser wavelength 1064 nm; laser power 350 mW) and Fourier transform infrared spectroscopy **(FT-IR**, Bruker RFS/100) spectrometry. For the measurement of electrical properties, the dried nanoparticles were uniaxially pressed in to disc-shaped pellets of 10 mm diameter using the isostatic pressure of 200 MPa. The pressed samples were sintered at 1273 K for 2 h. Top conductive electrodes were then deposited on both sides of sintered samples using silver paste. Dielectric constant and loss were measured at different temperatures and frequencies in the range of 298 K to 623 K and 100 Hz to 1 MHz respectively, using an impedance analyzer (4294A, Agilent, USA).

## Results and Discussion

### Structural properties

**Fig 1:**
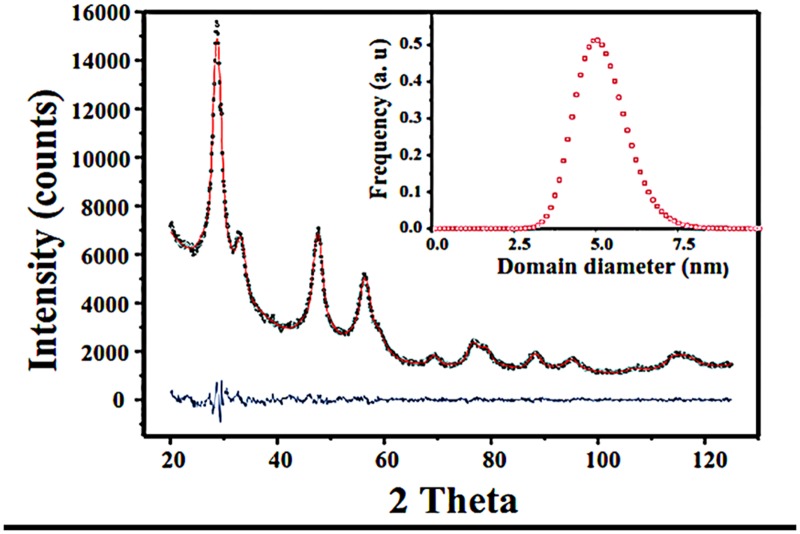
Graphical output of the WPPM modelling of CeO_2_ (black open squares are the calculated data, red continuous line the observed data, and the lower blue continuous line is the difference curve between observed and calculated profiles). In the inset is reported ceria crystalline domain size distribution.

XRD patterns shows cubic phase for prepared CeO_2_ nanoparticles (Fig 1). Furthermore, the whole powder pattern modelling (WPPM) method [27], taking advantage of the PM2K software [28,29], that allows for refinement of model parameters *via* a non-linear least squares routine, was employed for the micro structural analysis of CeO_2_. This procedure is considered to be a state-of-the-art methodology, and allows for the extraction of micro structural information from a diffraction pattern. With such a methodology, the experimental peaks are fitted without the use of any arbitrary analytical functions (*i*): the diffraction peak profile is the result of a convolution of instrumental and sample-related physical effects. As a consequence, the analysis is directly made in terms of physical models of microstructure and/or lattice defects.

Hence, with the WPPM method, aspects of microstructure such as the crystalline domain shape and size distribution can be accurately measured, with a method deeply superior to the estimations made by other frequently used integral breadth methods for line profile analysis (LPA)—*i*.*e*. the widely used Scherrer formula [30], or the Williamson–Hall method [31]. Actually, with the use of these quoted methods, it can be tricky to correctly extract integral breadths, because of the instrumental profile component, background and peak profile overlapping. Furthermore, additional sources of line broadening—*i*.*e*. domain size, and/or lattice strain—cannot be considered properly by LPA methods [32]. The instrumental contribution was obtained by modelling 14 *hkl* reflections from the NIST SRM 660b standard (LaB_6_), according to the Caglioti*et al*. relationship [33]. Afterward, CeO_2_ (fcc, space group (SG) *Fm3m*) was included in the WPPM modelling. The following parameters were refined: background (modelled using a 5^th^-order of the shifted Chebyshev polynomial function), peak intensities, specimen displacement, and lattice parameters. In this work, we assumed crystalline domains to be spherical, and distributed according to a lognormal size distribution. The WPPM results obtained for CeO_2_ samples are shown Table 1. The average crystalline domain diameter of prepared ceria was found to be 5.2 nm, with a very narrow size distribution with maxima centred at ~ 5 nm as depicted in inset of Fig 1. It is also interesting to note that no crystalline domains were detected below ~ 2.5 nm. All the crystalline domains were found in the range in between 2.5 to 7.5 nm.

**Table 1:** WPPM agreement factors, unit cell parameters and average crystalline domain diameter of CeO_2_.

Agreement factors			Unit cell parameters	Average crystalline domain diameter (nm)	Mode of the size distribution (nm)
*R*_wp_ (%)	*R*_exp_ (%)	*χ* ^2^	*a* = *b = c* (nm)		
2.85	1.81	1.57	0.5438(1)	5.2±0.1	5.0±0.1

### Scanning electron microscopy (SEM)

**Fig 2:**
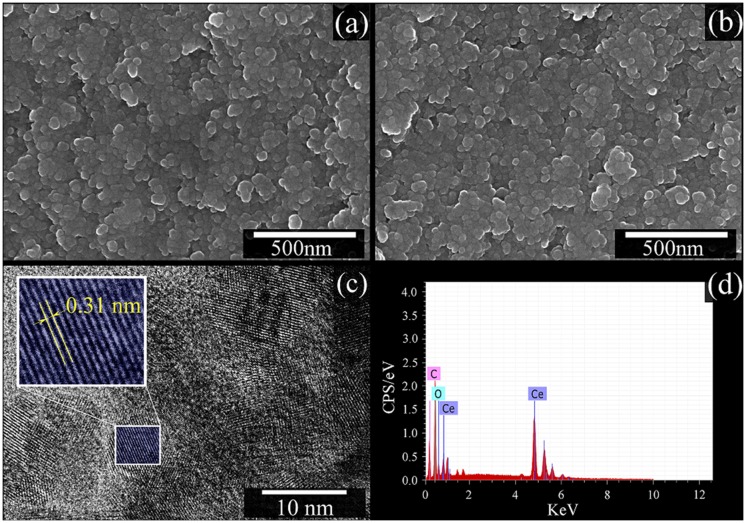
(a) and (b) SEM images, (c) HRTEM image and (d) EDS analysis of the CeO2 nanoparticle.

Fig 2a and 2b presents SEM images of the prepared CeO_2_ nanoparticles. The SEM images reveal synthesis of nano-sized CeO_2_ particles with an average diameter size of approximately ~25 nm (Fig 2a and 2b). Fig 2c shows HRTEM image obtained for prepared CeO_2_ nanoparticles. From HRTEM image analysis, the distance between neighbouring planes was measured equal to ~ 0.31 nm, which corresponds to (111) crystallographic plane of cubic CeO_2_ [34]. The corresponding EDS analysis for the sample is presented in Fig 2d. The presence of O and Ce elements was evident from the measured EDS spectra. No extra impurity was detected in EDS spectrum. The detected C signal was expected as a consequence of signals from conductive carbon tape used for sticking of the powder on the sample holder.

### Optical properties

**Fig 3:**
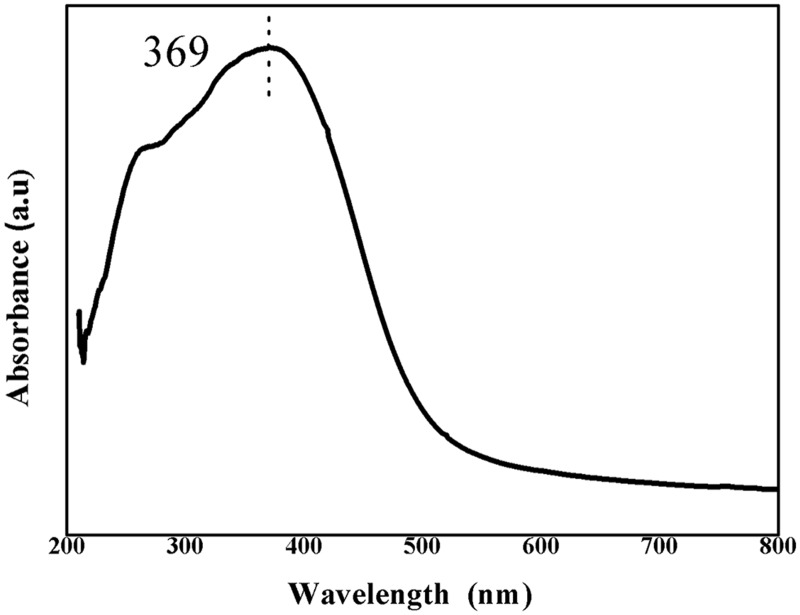
UV-Visible absorbance spectrum of prepared CeO_2_ nanoparticles.

Fig 3 shows the optical absorption spectra of prepared CeO_2_ nanoparticles in UV-visible range of electromagnetic wavelengths. In ultraviolet absorption process, the outer electrons of atoms or molecules absorb photons and undergo transitions to higher energy levels. Therefore, the obtained optical absorption spectrum can be used to calculate the energy band gap of the CeO_2_ material. UV-visible spectrum of the CeO_2_ nanoparticles shows a distinct absorption band at 369 nm. The optical band gap energy of CeO_2_ nanoparticles was calculated according to Eq (1) given by:

$$E_{bg}=\frac{1240}{\lambda }\left(eV\right)$$ (1)

Where, *λ* is the wavelength (nm) and *E*_bg_ is the optical band gap energy. The calculated optical band gap value of prepared CeO_2_ was found to be 3.36 eV, which is larger than that of 3.2 eV, reported in literature for bulk CeO_2_ [35]. The enhancement in optical band gap of CeO_2_ nanoparticle could be due to the quantum size effect and is consistent with literature report for CeO_2_ nanoparticles [36].

**Fig 4:**
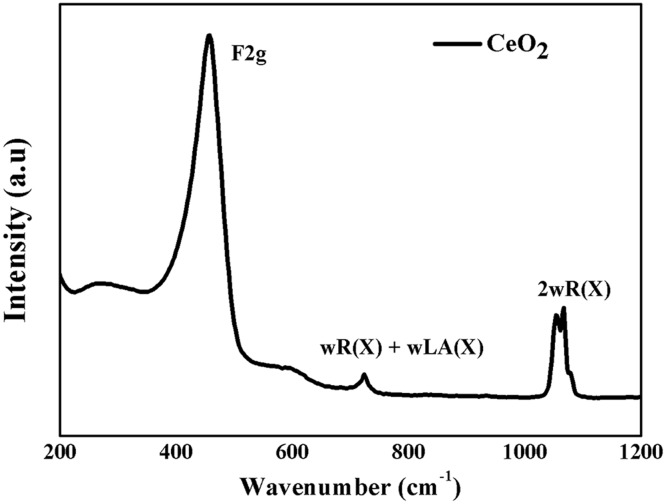
Raman spectrum of CeO_2_ nanoparticles.

CeO_2_ belongs to *O*_5h_ (Fm3m) space group with cubic fluorite structure. The first order Raman line at around 465 cm^-1^ attributed to triply degenerate Raman active optical phonon mode (*F*_2g_). The second order Raman spectrum, has nine phonon branches, with 45 possible phonon modes and are located at 580, 660, 880, 1030 and 1160 cm^-1^ for ωTO(X) + LA(X), ωR(X) + LA(X), ωLO + ωTO, 2ωR(X) and 2ωLO, respectively [16]. Fig 4 indicates the room temperature Raman spectrum of the CeO_2_ nanoparticles with the fluorite phase measured within a range of 200–1200 cm^-1^. A first order Raman peak (*F2g*) at 453 cm^-1^ and a two second order Raman peaks prominent at 717 and 1050 cm^-1^ were observed which are in good agreement with the previous reports [37].

**Fig 5:**
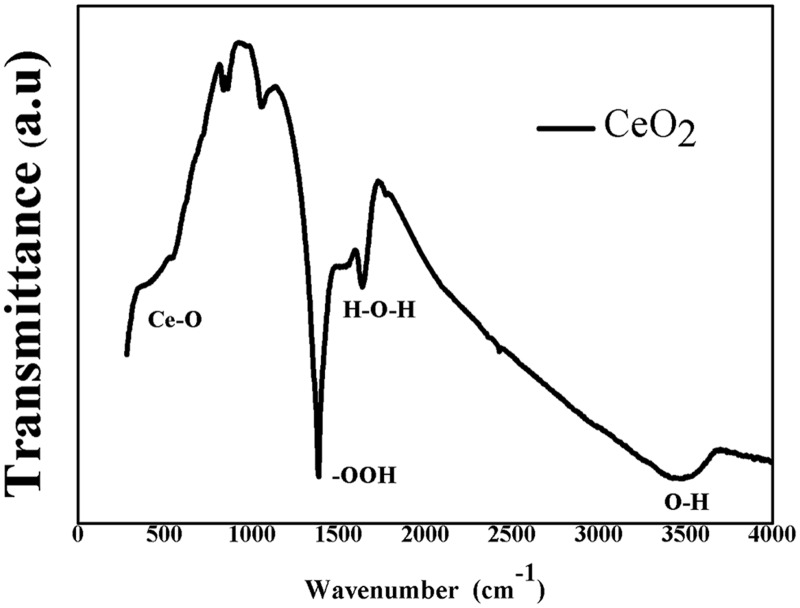
FT-IR spectrum of CeO_2_ nanoparticle.

Fig 5 shows the FTIR spectrum of prepared CeO_2_ nanoparticles. The prepared CeO_2_ nanoparticles reveals the presence of some absorption bands in the ranges from 400 cm^-1^ to 4000 cm^-1^, positioned at 450 cm^-1^, 1360 cm^-1^,1630 cm^-1^, and 3430 cm^-1^. The absorption band at 450 cm^-1^ corresponds to CeO_2_ stretching vibration. The signal at 1630 cm^-1^ is associated with the molecular H_2_O (H–O–H) bending frequency [38,39]. The broad adsorption band about 3430 cm^-1^ corresponds to hydrated and physically adsorbed water in the sample [40].

### Dielectric properties

**Fig 6:**
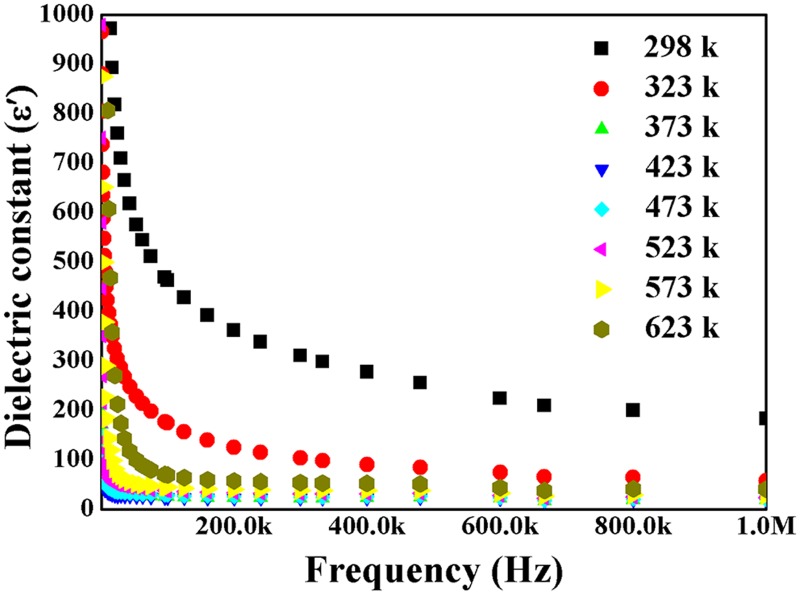
The dielectric constant of the CeO_2_ nanopowder versus frequency at various temperatures.

For the electrical characterization, namely dielectric constant (*ε*_*r*_) and dielectric loss (tan δ), the sintering conditions (1273 K for 2 h) that enabled achieving the high dense CeO_2_ ceramics consolidated from CeO_2_ nanoparticles were chosen. Fig 6 shows the frequency dispersion of *ε*_*r*_ values of prepared CeO_2_ ceramic, in the range from 1 kHz to 1 MHz measured at various temperatures from 298 K to 623 K. The high *ε*_*r*_ values at the low frequencies are because of interfacial or space charge polarisations. The *ε*_*r*_ values decreases with increasing frequency and reaches a constant value at higher frequencies. The obtained result is similar with works which were reported for dielectric ZnO nanocrystals [41,42]. Interfacial polarisation or space charge polarisation in CeO_2_ nanoparticles ceramic is due to the structural in-homogeneity because of large surface-to-volume ratio of nanoparticles. The external electric field causes the space charges move and trapped through the defects at the interfaces leading to formation of dipole moments. The space charge polarisation is dominant at low frequencies and Maxwell-Wagner dielectric dispersion theory is applicable to explain the dielectric behaviour of the sample [43–45]. Therefore, the grain boundaries effect is more pronounced at low frequencies. The electrons/holes hopping between positive and negative surface defect centres in the grain boundary region might constitute the dielectric polarisation at low frequencies. At higher frequencies, the dipoles rotational displacement results in orientational polarisation and with the increase of frequency, the electric dipoles in the material start to lag behind the applied electric field. However, the dielectric constant decreases exponentially and at higher frequencies, the dipoles fail to cope with rapid electric field variations. Therefore, a constant value was obtained for the dielectric constant at higher frequencies. It can also be noted from Fig 6 that, the dielectric constant is considerably decreased with increase in measuring temperature up to temperature of 373 K and thereafter start to increase a little bit with further increase of the temperature. This might be due to reduction of pore space volume between the particles, when the consolidated CeO_2_ nanoparticles compact was subjected to a high temperature sintering which causes larger grains growth at the expense of smaller grains and reduce of the energy barrier with rapid increase of the diffusion of atoms to another grain. Therefore, the sintered ceramic shows conductor type behaviour due to the increase of conductivity with increase of mobility.

**Fig 7:**
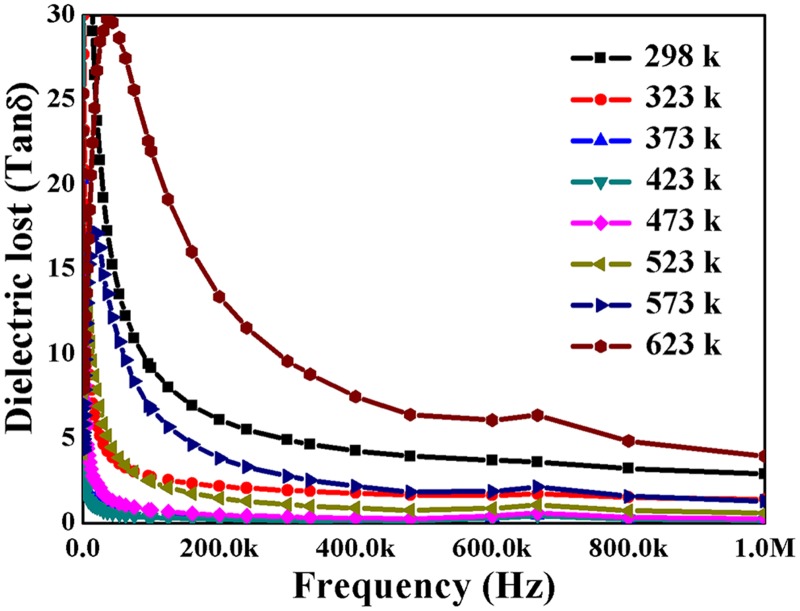
The dielectric loss CeO_2_ nanoparticles versus frequency of applied field at various temperatures.

The dielectric loss (tan δ) behaviours against frequency ranging from 1 kHz to 1MHz at various temperatures is shown in Fig 7. The tan δ values were found to decrease with increasing frequency due to the space charge polarization. Generally, the low loss is observed at higher frequencies, because the motion of domain wall is inhibited and magnetization is forced to change rotation. On the other hand the tan δ values were found to decrease with increasing temperatures till 473 K and then start to increase when measured at temperatures higher than473 K. The sintered CeO_2_ nanoparticles ceramic shows lower values of tan δ at 323 K, 373 K and 423 K when compared to values measured at 298 K (room temperature).

This indicates that the CeO_2_ nanoparticles dielectric ceramic can store more energy due to small loss and shows good dielectric behaviour at these temperatures. While, the tan δ values measured at 623 K were found to be high, revealing material cannot store energy at this temperature. The ac conductivity of the prepared CeO_2_ sample was further calculated using Eq (2) given by:

σac = ε' ε0ω tanδ (2)

**Fig 8:**
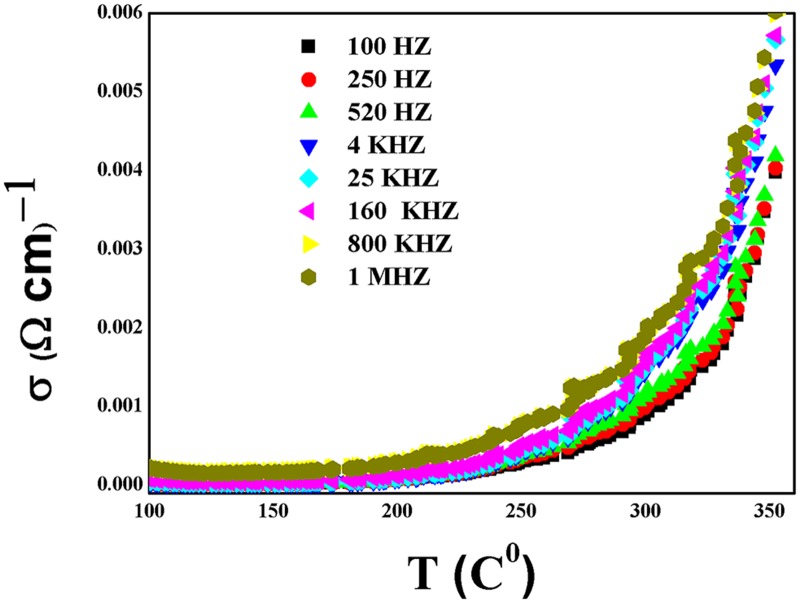
The variation of ac conductivity of CeO_2_ nanopartciles with temperature at different frequency.

Fig 8 shows the variation of ac conductivity calculated for prepared CeO_2_ nanoparticles ceramic with temperatures at different frequencies. As the temperature increases, the ac conductivity was found to be constant up to temperature of 450 K, and then increases with further increase in temperatures above 450 K. This behaviour could be due to thermally activated hopping of charge carriers between different localized states. Furthermore, the ac conductivity maximum value increases with increasing frequency, since the immigration of electrons is increased with increase in frequency.

## Conclusions

Low cost wet chemical method has been successfully established for the synthesis of CeO_2_ nanoparticles. The method was found to be convenient, rapid, and efficient in CeO_2_ nanoparticles synthesis. The average crystalline domain diameter from XRD analysis was found to be 5.2 nm, with a very narrow size distribution. The first order Raman line was obtained at around 465 cm^-1^ corresponding to triply degenerate Raman active optical phonon mode (*F*_2g_). The high values of dielectric constant for sintered CeO_2_ nanoparticles compact were observed in low frequencies regions due to structural in-homogeneity which was formed because of large surface-to-volume ratio of nanoparticles. Furthermore, the variations of dielectric constant values with measuring temperatures showed considerable decrease in dielectric constant values with increasing temperatures up to 373 K. Moreover, low loss values were found for prepared CeO_2_ samples at higher frequencies when measured at 323 K, 373 K and 423 K compared to loss values obtained at 298 K. The ac conductivity measured for prepared CeO_2_ samples was found to be constant with increase in temperature till 450 K, which further increases with increase in temperature above 450 K.
