# Correction: Dielectrical Properties of CeO_2_ Nanoparticles at Different Temperatures

**DOI:** 10.1371/journal.pone.0131851

**Published:** 2015-07-02

**Authors:** Reza Zamiri, Hossein Abbastabar Ahangar, Ajay Kaushal, Azmi Zakaria, Golnoosh Zamiri, David Tobaldi, J. M. F. Ferreira

There is an error in affiliation 3 for author Hossein Abbastabar Ahangar. The correct affiliation should be: Department of Chemistry, Faculty of Science, Najafabad Branch, Islamic Azad University, Najafabad, Isfahan, Iran.
